# Unveiling the Promise: A Comprehensive Review of Salpingectomy as a Vanguard for Ovarian Cancer Prevention

**DOI:** 10.7759/cureus.53088

**Published:** 2024-01-28

**Authors:** Priyal V Mundhada, Amey M Bakshi, Nikhil Thtipalli, Seema Yelne

**Affiliations:** 1 Medicine, Jawaharlal Nehru Medical College, Datta Meghe Institute of Higher Education and Research, Wardha, IND; 2 Surgery, Jawaharlal Nehru Medical College, Datta Meghe Institute of Higher Education and Research, Wardha, IND; 3 Nursing, Shalinitai Meghe College of Nursing, Datta Meghe Institute of Higher Education and Research, Wardha, IND

**Keywords:** reproductive health, ethical considerations, clinical trials, tubal hypothesis, ovarian cancer prevention, salpingectomy

## Abstract

This comprehensive review explores the potential of salpingectomy as a groundbreaking strategy for the prevention of ovarian cancer. The discussion encompasses the biological rationale behind salpingectomy, emphasizing its foundation in the tubal hypothesis, which posits the fallopian tubes as a possible origin site for certain ovarian cancers. Ongoing clinical trials and observational studies provide evolving evidence supporting the safety and efficacy of salpingectomy, particularly in high-risk populations. The procedure's ethical considerations, including its impact on fertility and equitable access, are thoroughly examined. Implications for clinical practice underscore the importance of informed decision-making, risk-benefit assessments, and the integration of emerging evidence into reproductive health discussions. Looking ahead, the future landscape of ovarian cancer prevention involves continued research, technological innovations, and collaborative efforts to ensure a holistic and evidence-based approach. The goal is to forge a future where ovarian cancer is not only treatable but also preventable, with salpingectomy potentially playing a pivotal role in this transformative journey.

## Introduction and background

Ovarian cancer is a malignancy that arises from the ovarian tissue and is notorious for its asymptomatic progression in the early stages, contributing to delayed diagnoses and reduced treatment efficacy. According to global cancer statistics, ovarian cancer ranks as the eighth most common cancer among women and is responsible for a significant proportion of cancer-related deaths. Understanding the epidemiology, risk factors, and challenges associated with ovarian cancer is crucial for framing effective preventive measures [1].

The impact of ovarian cancer on women's health and well-being cannot be overstated. Beyond the physical toll, the emotional and socioeconomic repercussions reverberate through affected individuals and their communities. Ovarian cancer prevention holds the promise of not only reducing the incidence of this devastating disease but also alleviating the burden it places on healthcare systems and society at large. Exploring avenues for prevention becomes imperative in the broader context of women's health and cancer control [2].

Salpingectomy, the surgical removal of the fallopian tubes, has emerged as a potential game-changer in the landscape of ovarian cancer prevention. The fallopian tubes, once considered merely conduits for egg transport, are now implicated in the origin of some ovarian cancers. Understanding the biological underpinnings of this connection sets the stage for exploring salpingectomy as a prophylactic measure against ovarian malignancies [3].

This comprehensive review aims to synthesize existing knowledge, scrutinize pertinent clinical studies, and assess the evolving role of salpingectomy in ovarian cancer prevention. By delving into the mechanisms, clinical outcomes, and challenges associated with salpingectomy, we aim to provide clinicians, researchers, and policymakers with a nuanced understanding of this surgical intervention's potential benefits and considerations. Through this exploration, we aspire to contribute to the ongoing discourse on preventive strategies, ultimately striving toward a future where ovarian cancer is treatable and preventable.

## Review

Understanding ovarian cancer

Epidemiology and Risk Factors

Ovarian cancer is a relatively rare cancer, accounting for only 1.3% of new cancer diagnoses in 2019 [4]. However, it is a deadly cancer, with only 47.6% of patients surviving for five years or longer [4]. The American Cancer Society estimates that in the United States, in 2023, about 19,710 women will receive a new diagnosis of ovarian cancer, and about 13,270 women will die from ovarian cancer [4]. The incidence rate of ovarian cancer has been slowly falling over the past 20 years, with a 1-2% annual decrease from 1990 [5]. The risk of ovarian cancer is one in 78 for a woman during her lifetime, and the lifetime chance of dying from ovarian cancer is about one in 108 [5]. The risk of ovarian cancer is higher for women who have inherited the risk from their mother or father [4]. Other risk factors for ovarian cancer include age. About half of the women who are diagnosed with ovarian cancer are 63 years or older [5]. In terms of ethnicity, ovarian cancer is more common in White women than Black women. [5].

Challenges in Early Detection

Early detection of ovarian cancer is crucial for high cure rates. Still, it has been challenging due to the disease's low prevalence, specificity, and high rates of false positives [6]. Despite the development of new biomarkers and imaging techniques, an effective strategy for early detection has yet to be developed [6]. The UK Collaborative Trial of Ovarian Cancer Screening (UKCTOCS) trial, which tested a two-step screening strategy, found no reduction in mortality despite increased detection of early stage disease [7]. This highlights the need for further research and development of more effective approaches for early detection of ovarian cancer.

Salpingectomy: a novel approach

Salpingectomy, derived from the Greek words "salpinx" (tube) and "ektome" (cutting out), is a surgical procedure involving the removal of one or both fallopian tubes. Historically perceived primarily as a method for contraception or as part of fertility-preserving surgeries, salpingectomy has recently garnered attention for its potential role in ovarian cancer prevention. The procedure can be performed through various surgical approaches with nuances and implications [8].

The roots of salpingectomy trace back to early surgical practices, where removal of the fallopian tubes was primarily associated with addressing reproductive health concerns. Over time, evolving insights into the anatomical and physiological aspects of the female reproductive system have contributed to a broader understanding of the fallopian tubes' role in gynecological health. Historical milestones in the evolution of salpingectomy as a therapeutic and preventive measure reflect the ongoing refinement of surgical techniques and a deepening comprehension of its potential implications [9].

The rationale for employing salpingectomy as a preventive strategy against ovarian cancer stems from emerging evidence suggesting the fallopian tubes as a possible site of origin for certain types of ovarian malignancies. The "tubal hypothesis" proposes that precursor lesions and early stage cancers may arise in the fallopian tubes before spreading to the ovaries. By removing these potentially problematic tissues, salpingectomy aims to interrupt the progression of ovarian cancer at an early stage, offering a prophylactic approach to mitigate the risk of future malignancy [10].

Types of Salpingectomy Procedures

Unilateral salpingectomy: Unilateral salpingectomy involves the surgical removal of one fallopian tube while preserving the other. This procedure is often considered in cases where fertility preservation is a primary concern, especially in women who may wish to conceive in the future. Additionally, unilateral salpingectomy may be performed when pathologies, such as a cyst or tumor, are localized to one fallopian tube. This targeted approach allows the removal of the affected tube while retaining the potential for natural conception and reproductive function through the preservation of the contralateral fallopian tube [11].

Bilateral salpingectomy: Bilateral salpingectomy is characterized by the complete removal of both fallopian tubes. This surgical intervention is frequently undertaken as part of elective procedures, especially when there is no immediate concern for fertility preservation. Additionally, bilateral salpingectomy is performed in cases where bilateral pathology, such as the presence of cancerous lesions or a heightened risk of ovarian cancer, is identified. By removing both fallopian tubes, this approach aims to comprehensively address potential risks associated with the fallopian tubes while potentially reducing the risk of certain ovarian cancers [12].

Total salpingectomy: Total salpingectomy involves the comprehensive removal of the entire fallopian tube, encompassing both the fimbrial end and its connection to the uterus. This thorough approach is often employed when there is a heightened risk of ovarian cancer or when other gynecological conditions necessitate complete removal. By excising the entire fallopian tube, including its connection to the uterus, total salpingectomy aims to eliminate any potential source of pathology or cancerous development within the fallopian tubes [13].

Partial salpingectomy: Partial salpingectomy entails the removal of a specific portion of the fallopian tube. This surgical approach is typically indicated when preserving part of the tube is deemed appropriate. For example, in cases with a benign condition or a localized issue within the fallopian tube, removing only the affected portion allows for retaining the remaining healthy section. This approach is often considered when balancing the need for intervention to preserve reproductive function or mitigate potential side effects associated with the complete removal of the fallopian tube [14].

Mechanisms and theoretical framework

Fallopian Tube and Ovarian Connection

Understanding the intricate relationship between the fallopian tubes and the development of ovarian cancer forms the basis for the exploration of salpingectomy as a preventive measure. The fallopian tubes, traditionally recognized for their role in facilitating egg transport, have come under scrutiny due to emerging evidence suggesting their involvement in the early stages of certain ovarian cancers. The anatomical proximity and shared peritoneal environment between the fallopian tubes and ovaries create a microenvironment that may contribute to the initiation and progression of malignancies [15].

Proposed Mechanisms for Ovarian Cancer Prevention

Interrupting tubal carcinogenesis: The rationale behind salpingectomy as a preventive strategy for ovarian cancer lies in the "tubal hypothesis." According to this hypothesis, serous ovarian cancers, the most prevalent subtype, may originate in the fallopian tubes. Salpingectomy aims to interrupt this potential pathway by surgically removing the fallopian tubes. By doing so, the procedure eliminates the site where precursor lesions or early stage cancers may develop. Based on understanding the anatomical and histological connection between the fallopian tubes and ovaries, this intervention seeks to halt the progression of ovarian malignancies at their inception [16].

Disrupting inflammatory processes: Inflammation within the fallopian tubes has been identified as a contributing factor to the development of ovarian cancer. Salpingectomy emerges as a potential risk mitigation strategy by eliminating the source of inflammation. The surgical removal of the fallopian tubes aims to disrupt the inflammatory processes in this anatomical region, thereby reducing the likelihood of malignant transformation. This approach represents a targeted intervention to interrupt a critical biological pathway associated with the initiation and progression of ovarian cancers [17].

Altering hormonal and microenvironmental factors: Removing fallopian tubes through salpingectomy introduces the possibility of altering hormonal and microenvironmental factors within the pelvic cavity. The fallopian tubes play a role in hormonal signaling and contribute to the local environment in the pelvic region. Salpingectomy is theorized to create a favorable shift in these factors, potentially creating an unfavorable milieu for the development and progression of ovarian malignancies. This multifaceted impact underscores the broader systemic effects that the procedure may exert on the hormonal balance and microenvironment within the pelvic cavity, potentially influencing the risk landscape for ovarian cancer [9].

Supporting Evidence From Preclinical Studies

Preclinical studies have provided valuable insights into the mechanisms of ovarian cancer metastasis and potential therapeutic targets. Research has identified various biochemical pathways and molecular mechanisms involved in ovarian cancer progression. For instance, the role of metastasis suppressor genes, such as *Nm23*, has been investigated, highlighting their potential as therapeutic targets [18]. The molecular mechanisms of cell apoptosis, autophagy, RNA and DNA lesion, reactive oxygen species damage, and multiple-drug resistance have also been the research focus, offering potential avenues for developing anti-ovarian cancer therapies [19].

Furthermore, studies have explored the role of the tumor microenvironment in the abdominal cavity in ovarian cancer progression. Research has suggested that normalizing the tumor microenvironment may indirectly suppress ovarian cancer cells, presenting a novel therapeutic approach that warrants further investigation [20]. Moreover, the outcomes from opportunistic salpingectomy for ovarian cancer prevention have been studied, with findings indicating that this approach may lead to significantly fewer serous ovarian cancers than expected. This suggests the potential of opportunistic salpingectomy as a preventive strategy for high-grade serous ovarian cancers [21]. Preclinical studies have contributed to our understanding of the mechanisms of ovarian cancer metastasis and have identified potential therapeutic targets, including metastasis suppressor genes, molecular pathways, and the tumor microenvironment. These findings hold promise for developing novel anti-ovarian cancer therapies and preventive strategies.

Clinical studies and observational data

Overview of Relevant Clinical Trials

The RESPONSE trial is an observational, multinational cohort study that collected real-world medical record data from eight Western countries on the diagnostic workup, clinical outcomes, and treatment strategies for women with newly diagnosed advanced high-grade serous or endometrioid ovarian cancer [22]. A clinical trial at the Mayo Clinic aims to identify candidate biomarkers for early detection and chemotherapy treatment response in high-grade serous cancer (HGSC) [23]. The National Cancer Institute (NCI) supports clinical trials studying new, more effective ways to detect and treat ovarian cancer. The NCI website provides information on clinical trials for ovarian cancer [24]. The RESPONSE and the clinical trials at Mayo Clinic are relevant observational and interventional studies, respectively, that aim to improve the diagnosis and treatment of ovarian cancer. The NCI also supports clinical trials studying new and more effective ways to detect and treat ovarian cancer.

Comparative Studies: Salpingectomy Versus Other Preventive Measures

The current landscape of performing bilateral salpingectomy for ovarian cancer prevention is promising. A systematic review of 158 articles found that salpingectomy was associated with an ovarian cancer risk reduction of approximately 80% [3]. With widespread implementation, salpingectomy has the potential to reduce ovarian cancer mortality in the U.S. by an estimated 15% [3]. Studies have demonstrated that salpingectomy was safe, cost-effective, and not associated with earlier menopause onset [3]. Opportunistic salpingectomy, the removal of fallopian tubes during hysterectomy or instead of tubal ligation without removal of ovaries, has been recommended to prevent ovarian cancer, particularly serous ovarian cancer [21]. However, the effectiveness of opportunistic salpingectomy is still being studied, and prospective studies to demonstrate long-term survival outcomes and feasibility in nongynecological surgical procedures are warranted [21]. Overall, bilateral salpingectomy for ovarian cancer prevention appears to be safe, feasible, and potentially cost-effective, with the potential to reduce ovarian cancer mortality significantly.

Long-Term Follow-Up Data

Long-term follow-up data from the UKCTOCS revealed that after a median follow-up of 16.3 years, ovarian cancer screening did not significantly reduce mortality compared to usual care [25]. Additionally, a study on the use of poly ADP-ribose polymerase inhibitor maintenance therapy in ovarian cancer patients showed no difference in overall survival between patients who received the therapy and those who did not, based on long-term follow-up data [26]. Furthermore, the RESPONSE trial, an observational, multinational cohort study, aimed to describe the treatment strategies and outcomes for women with newly diagnosed advanced high‐grade serous or endometrioid ovarian cancer. The study collected real-world medical record data on the diagnostic workup, clinical outcomes, and treatment strategies for ovarian cancer from eight Western countries [22]. In addition, a clinical trial at the Mayo Clinic aims to identify candidate biomarkers for early detection and chemotherapy treatment response in HGSC. The study consists of two parts: Part A, which focuses on dose regimen finding, and Part B, which involves dose expansion to study potential therapeutic doses [23].

Safety and feasibility

Surgical Techniques and Approaches

Laparoscopic salpingectomy: Laparoscopic salpingectomy represents a minimally invasive surgical technique wherein a laparoscope is inserted through small incisions, allowing for the visualization and removal of the fallopian tubes. This method offers distinct advantages over traditional open procedures, including shorter hospital stays, decreased postoperative pain, and a quicker recovery. By leveraging the precision of laparoscopic instruments and minimizing the procedure's invasiveness, laparoscopic salpingectomy has become a preferred approach for clinicians seeking to balance effective treatment with reduced patient recovery times [27].

Robotic-assisted salpingectomy: Robotic-assisted salpingectomy integrates laparoscopy with robotic technology to enhance the surgeon's precision and control during the procedure. This approach shares similarities with laparoscopic techniques but employs robotic arms operated by the surgeon to perform intricate maneuvers. Robotic-assisted salpingectomy is particularly advantageous in situations where fine-tuned movements are required. While offering benefits similar to laparoscopy, such as smaller incisions and faster recovery, the robotic approach provides an additional layer of dexterity, making it an attractive option for complex surgeries that demand high precision [28].

Open salpingectomy: Open salpingectomy involves traditional open surgery, necessitating a larger incision that provides direct access to the fallopian tubes. Despite being associated with longer recovery times and increased postoperative discomfort compared to minimally invasive approaches, open salpingectomy may be deemed necessary in specific clinical scenarios. Instances where intricate maneuvers are challenging or when dealing with complex pathologies may warrant using an open approach. The decision to opt for open salpingectomy is often guided by the surgeon's judgment and the unique requirements of the individual case, emphasizing the importance of tailoring the surgical approach to the specific clinical context [29].

Complication Rates

Intraoperative complications: Intraoperative complications are potential challenges that may arise during the surgical procedure. These complications can include injury to surrounding structures, bleeding, or difficulties in accessing the fallopian tubes. The frequency and severity of intraoperative complications are pivotal considerations for surgical safety. Surgeons must navigate anatomical complexities carefully, and any inadvertent injuries or complications during this phase can impact immediate and long-term patient outcomes. Rigorous attention to surgical techniques, expertise, and thorough preoperative planning is essential to minimize the risk of intraoperative complications and ensure the overall safety of the salpingectomy procedure [30].

Postoperative complications refer to adverse events that may occur after the salpingectomy procedure. Common postoperative complications include infections, hematoma formation (accumulation of blood outside blood vessels), and adverse reactions to anesthesia. Monitoring and managing these immediate complications are critical for the patient's well-being during the early recovery. Additionally, evaluating long-term complications is essential, particularly concerning their potential impacts on reproductive and overall health. Understanding the incidence and nature of postoperative complications contributes to refining surgical protocols, enhancing patient care, and informing preoperative counseling to ensure a comprehensive approach to the safety and well-being of individuals undergoing salpingectomy [31].

Patient Acceptance and Satisfaction

Informed consent and counseling: Informed consent and comprehensive counseling are the cornerstones of ethical and patient-centered salpingectomy care. Ensuring that individuals considering the procedure are well-informed about its rationale, potential benefits, and associated risks is paramount. This includes a detailed discussion about the impact of salpingectomy on fertility, hormonal status, and overall well-being. A clear and understandable overview enables patients to make informed decisions aligned with their values and reproductive goals. Preoperative counseling, therefore, serves as a crucial step in establishing trust, respecting patient autonomy, and fostering a collaborative approach between healthcare providers and individuals undergoing salpingectomy [10].

Postoperative recovery and follow-up: The patient experience extends beyond the operating room, and considerations for postoperative recovery are vital in optimizing overall satisfaction. Managing postoperative pain, facilitating a smooth recovery process, and enabling the timely restoration of normal activities contribute to patient well-being. Additionally, establishing effective follow-up care is essential. This involves monitoring potential complications, addressing patient concerns, and providing ongoing support during recovery. A comprehensive and supportive postoperative care plan enhances patient satisfaction, promotes a positive perception of the procedure, and reinforces the patient-provider relationship [32].

Long-term impact on reproductive health: Assessing the long-term impact of salpingectomy on reproductive health, particularly in premenopausal women, is critical for understanding the broader implications of the procedure. Long-term follow-up studies are pivotal in gauging patient satisfaction and reproductive outcomes post-salpingectomy. By tracking fertility, hormonal changes, and overall reproductive health, these studies provide essential insights into the acceptability of salpingectomy as a preventive measure. Understanding the long-term consequences ensures that healthcare providers can offer nuanced and comprehensive guidance to individuals considering or undergoing salpingectomy, addressing their holistic health needs and promoting informed decision-making [33].

Challenges and controversies

Ethical Considerations

Impact on fertility and informed consent: The potential impact of salpingectomy on fertility introduces a nuanced ethical consideration, requiring a delicate balance between the imperative of cancer prevention and the preservation of reproductive choices. Recognizing the potential implications for fertility, hormonal status, and overall reproductive health, obtaining informed consent becomes a cornerstone of ethical practice. Comprehensive preoperative counseling ensures that patients are fully informed about the potential consequences of salpingectomy on their reproductive options, fostering autonomy in decision-making and aligning the procedure with individual values and goals [34].

Elective versus therapeutic procedures: Performing salpingectomy as an elective, prophylactic measure without diagnosed pathology prompts ethical reflections. Clinicians are tasked with carefully weighing the potential benefits of cancer prevention against the fundamental principle of avoiding unnecessary interventions. Ensuring that elective salpingectomy is ethically justified demands a robust evaluation of individual risk profiles, informed decision-making, and adherence to established ethical guidelines. Ethical considerations are paramount in guiding clinicians to make informed and justifiable decisions about the appropriateness of salpingectomy as a preventive measure without a confirmed diagnosis [35].

Equitable access and informed decision-making: Ethical imperatives extend to ensuring equitable access to information and healthcare resources related to salpingectomy, preventing disparities in informed decision-making. Ethical concerns arise if specific populations face barriers to understanding the procedure or lack access to necessary healthcare resources, potentially leading to disparities in preventive care. Addressing these disparities aligns with principles of justice and autonomy, emphasizing the ethical obligation to provide comprehensive information and support to all individuals, regardless of background or socioeconomic status, enabling them to make informed decisions about their reproductive and preventive health [36].

Potential Drawbacks and Limitations

Limited efficacy across ovarian cancer subtypes: The emphasis on high-grade serous ovarian cancers in the tubal hypothesis prompts critical questions about the universal efficacy of salpingectomy across various ovarian cancer subtypes. The uncertainty regarding its effectiveness for other histological subtypes underscores the need for further research to delineate the range of ovarian malignancies impacted by salpingectomy. The current ambiguity raises concerns about the generalizability of salpingectomy as a preventive strategy, highlighting the importance of expanding research efforts to encompass a broader spectrum of ovarian cancer subtypes [37].

Optimal timing and applicability: Determining the optimal timing for salpingectomy in the context of ovarian cancer prevention poses an ongoing challenge. Understanding when the procedure is most beneficial, especially for significant events, such as childbirth or menopause, requires dedicated investigation. The complex interplay between timing and applicability demands comprehensive research to guide healthcare providers and individuals in making informed decisions about the most opportune junctures for undergoing salpingectomy. This research would contribute to a nuanced understanding of the procedure's impact across different life stages [38].

Surgical risks and complications: Like any surgical intervention, salpingectomy has inherent risks. Potential complications, including bleeding, infection, or inadvertent damage to adjacent structures, underscore the importance of understanding and mitigating these risks. A thorough assessment of the safety profile of salpingectomy is imperative for informed decision-making by healthcare providers and patients. Ongoing efforts to refine surgical techniques and minimize risks enhance salpingectomy's overall feasibility and safety as a preventive measure. Addressing these concerns is integral to ensuring that the potential benefits of ovarian cancer prevention are weighed against the associated surgical risks in a well-informed and ethical manner [39].

Unanswered Questions and Areas for Future Research

Long-term follow-up studies: The current scarcity of long-term follow-up data presents a significant gap in our understanding of the sustained preventive effects of salpingectomy. Robust and extended studies are imperative to evaluate the procedure's impact on ovarian cancer incidence over an extended period. These studies contribute to assessing the longevity of preventive effects and identifying potential late-onset complications or unintended consequences associated with salpingectomy. Comprehensive long-term data are essential for refining clinical guidelines and ensuring salpingectomy's continued safety and efficacy as a preventive strategy [40].

Reproductive outcomes: Comprehensive research focusing on the reproductive outcomes of women undergoing salpingectomy is crucial for a holistic understanding of the procedure's impact. This includes assessing fertility, hormonal changes, and the potential implications for individuals seeking future pregnancies. Studies examining the reproductive experiences of women post-salpingectomy contribute valuable insights into the nuanced considerations surrounding the procedure, thereby aiding healthcare providers and individuals in making informed decisions aligned with their family planning goals [41].

Effectiveness in high-risk populations: Understanding the effectiveness of salpingectomy in high-risk populations, such as individuals with a strong family history or known genetic mutations, is an essential area that requires targeted investigation. High-risk individuals often necessitate tailored preventive strategies, and understanding the efficacy of salpingectomy in this specific context is vital. Research in this domain contributes not only to the evidence base supporting the use of salpingectomy but also to the development of personalized approaches for individuals with an elevated risk of ovarian cancer [42].

Comparative effectiveness studies: Comparative effectiveness studies that assess salpingectomy compared to other preventive measures, such as tubal ligation or risk-reducing medications, offer valuable insights into each approach's relative benefits and drawbacks. These studies provide a basis for evidence-based decision-making, allowing healthcare providers and individuals to weigh the pros and cons of different preventive strategies. Comparative effectiveness research contributes to the refinement of clinical guidelines, ensuring that the selection of preventive measures aligns with individualized risk profiles and preferences [43].

Health policy implications

Integration Into Ovarian Cancer Prevention Guidelines

Guideline development: Health policy initiatives should prioritize collaborative efforts involving healthcare professionals, researchers, and policymakers to develop comprehensive guidelines for implementing salpingectomy as an ovarian cancer prevention strategy. Based on the best available evidence, these guidelines should address indications, contraindications, and procedural details. Regular updates to guidelines are crucial to reflect advancements in research and evolving clinical practices. A multidisciplinary approach is essential to ensure that guidelines are evidence-based, consider ethical considerations, and provide clear recommendations to guide healthcare providers in the safe and effective implementation of salpingectomy [44].

Inclusion in screening programs: Integrating salpingectomy into national or regional cancer screening programs is a pivotal step in promoting widespread access to preventive measures for individuals at risk of ovarian cancer. Health policy initiatives should focus on incorporating salpingectomy as a viable option within existing screening frameworks. Achieving this involves developing educational campaigns to inform healthcare providers and the public about the potential benefits of salpingectomy in ovarian cancer prevention. This proactive approach ensures that individuals are well-informed about available preventive options, facilitating informed decision-making and contributing to the overall success of cancer prevention strategies [45].

Access to Salpingectomy as a Preventive Measure

Education and awareness campaigns: Health policy initiatives should actively support public awareness campaigns to inform individuals about the potential benefits of salpingectomy as a preventive measure for ovarian cancer. Disseminating information through diverse channels, including healthcare providers, community outreach programs, and online resources, is crucial. Policies should encourage the development of educational materials that are accessible and culturally sensitive, fostering a comprehensive understanding of salpingectomy and empowering individuals to make informed decisions about their reproductive health [46].

Insurance coverage: Health policies should advocate for inclusive insurance coverage encompassing salpingectomy as a preventive measure. Clear guidelines on reimbursement for the procedure can incentivize healthcare providers to offer salpingectomy as part of comprehensive reproductive health services. Policies should address potential barriers to coverage, ensuring individuals have affordable access to this preventive option. By aligning insurance policies with the promotion of salpingectomy, health policy initiatives reduce barriers to preventive care [47].

Inclusion in public health programs: Integrating salpingectomy into public health programs, particularly those focused on women's health, is instrumental in ensuring widespread accessibility to the procedure. Health policies should encourage collaboration between public health agencies and healthcare institutions to streamline the inclusion of salpingectomy within existing public health frameworks. This collaborative effort facilitates the development of targeted initiatives, reaching diverse populations and addressing potential disparities in access. By incorporating salpingectomy into public health programs, health policies contribute to implementing evidence-based preventive strategies on a broader scale [48].

Economic Considerations

Cost-benefit analyses: Health policy should actively support and facilitate rigorous cost-benefit analyses to comprehensively evaluate the economic impact of integrating salpingectomy into preventive care strategies. These analyses should assess short-term and long-term costs, considering procedure expenses, potential complications, and downstream savings related to ovarian cancer prevention. The findings from such analyses can inform decision-makers about the economic viability of adopting salpingectomy as a preventive measure, aiding in developing evidence-based health policies [49].

Resource allocation: Health systems must carefully consider resource allocation when implementing salpingectomy as a preventive measure. This involves evaluating the costs associated with training healthcare professionals, establishing necessary infrastructure, and providing resources for the procedure. Policies should guide health systems in efficiently allocating resources to ensure the successful integration of salpingectomy into preventive care frameworks. Clear guidelines on resource allocation contribute to the sustainable implementation of salpingectomy and promote effective utilization of healthcare resources [50].

Public health investments: Health policies should advocate for public health investments in research and the implementation of salpingectomy as a preventive measure. Allocating funds for studies on the long-term effectiveness, safety, and impact on overall healthcare expenditures is crucial. These investments support ongoing research efforts, ensuring the evidence base for salpingectomy remains robust. Moreover, financial support for implementing salpingectomy programs, including educational initiatives and infrastructure development, is essential for successfully integrating this preventive measure into broader public health strategies [51].

Future directions and research agenda

Ongoing and Planned Clinical Trials

Identification of ongoing trials: Health policies should prioritize a systematic review of ongoing clinical trials focused on salpingectomy to gain insights into the current research landscape. This comprehensive review should encompass trials investigating the preventive effects of salpingectomy, comparing different surgical techniques, and assessing long-term outcomes. The findings from ongoing trials provide valuable information for shaping evidence-based health policies and guiding future research directions in ovarian cancer prevention [52].

Evaluation of trial designs: Analyzing the designs of ongoing trials is crucial for researchers and policymakers to assess the robustness of evidence that will emerge from these studies. This evaluation should include sample sizes, patient populations, and outcome measures. Understanding the intricacies of trial designs informs the interpretation of trial results and helps policymakers make informed decisions about the potential integration of salpingectomy into preventive care strategies. Additionally, insights from evaluating trial designs contribute to refining methodologies in future research endeavors [53].

Translation of preclinical findings: Ongoing trials should be aligned with and build upon preclinical findings to ensure a translational approach from bench to bedside. This alignment enhances the relevance of clinical research, allowing for a seamless integration of preclinical insights into the design and objectives of clinical trials. By ensuring that ongoing trials are rooted in the foundational knowledge derived from preclinical studies, policymakers can foster a more comprehensive understanding of salpingectomy's preventive potential. This translational approach contributes to developing evidence-based health policies grounded in the continuum of scientific discovery [54].

Emerging Technologies and Innovations

Advancements in surgical technology: Health policies should emphasize the continuous monitoring and incorporation of advancements in surgical technology to enhance the precision and safety of salpingectomy. This includes staying abreast of developments in robotic-assisted procedures, novel laparoscopic techniques, and other innovative approaches. Policies encouraging research and the adoption of state-of-the-art surgical technologies contribute to optimizing surgical outcomes, minimizing complications, and improving the overall patient experience [55].

Molecular and genetic discoveries: Ongoing exploration of molecular and genetic factors associated with ovarian cancer is pivotal for uncovering new biomarkers or targets for intervention. Health policies should support the integration of these discoveries into preventive strategies, including salpingectomy. By aligning policies with the evolving molecular and genetic research landscape, policymakers can foster the development of personalized approaches to ovarian cancer prevention. This integration ensures that preventive measures, such as salpingectomy, are continually refined based on the latest scientific insights [56].

Imaging modalities: Health policies should encourage research efforts focused on improving imaging modalities for the early detection of ovarian abnormalities. Advancements in imaging techniques can be crucial in refining the selection of candidates for salpingectomy, enabling a more targeted and personalized approach to preventive care. Policies that promote the integration of advanced imaging modalities into diagnostic and preventive pathways contribute to enhancing the overall effectiveness of salpingectomy as a preventive measure for ovarian cancer [57].

Collaborative Efforts in Ovarian Cancer Prevention

International collaborations: International collaborations in ovarian cancer prevention are pivotal in fostering a collective and global approach to addressing this complex health challenge. Collaborative initiatives on an international scale facilitate the pooling of resources, data, and expertise from diverse healthcare systems and populations. Shared databases and collaborative studies enhance research findings' statistical power and generalizability and promote a unified and comprehensive understanding of ovarian cancer prevention. By transcending geographical boundaries, international collaborations contribute to developing evidence-based health policies that can be applied globally, ensuring that a broad and diverse range informs preventive strategies of experiences and data [58].

Interdisciplinary research teams: The multifaceted nature of ovarian cancer and its prevention necessitates a collaborative and interdisciplinary approach. Building research teams that bring together experts from diverse fields, including gynecology, oncology, genetics, and public health, fosters a holistic understanding of the disease. This collaborative model integrates diverse perspectives, methodologies, and expertise, creating a comprehensive framework for investigating and addressing ovarian cancer prevention. Interdisciplinary research teams are better equipped to navigate the complexity of genetic, environmental, and healthcare factors contributing to ovarian cancer, leading to more nuanced and effective preventive strategies. This approach is essential for the development of evidence-based health policies that reflect the interconnected nature of ovarian cancer and its prevention [59].

Patient advocacy and involvement: Involving patient advocacy groups in planning and implementing research initiatives is paramount for ensuring that the patient perspective is central to developing and accessing preventive strategies. Patient advocacy and involvement serve as crucial mechanisms for incorporating the lived experiences and priorities of individuals affected by ovarian cancer into the research process. Engaging with patients helps address concerns, enhance informed decision-making, and prioritize research areas aligning with patient needs. By actively involving patients in the research continuum, from study design to implementation and dissemination of results, health policies can be developed with a heightened sensitivity to ovarian cancer prevention's practical and emotional aspects. This patient-centric approach contributes to developing more compassionate and effective health policies that resonate with the experiences and preferences of those at the heart of the matter [60].

## Conclusions

In conclusion, the exploration of salpingectomy as a prophylactic measure against ovarian cancer has yielded significant insights into its biological rationale, clinical evidence, and ethical considerations. The foundation of this preventive strategy lies in the tubal hypothesis, positing that the fallopian tubes may serve as a site of origin for certain ovarian cancers. Ongoing clinical trials and observational studies have contributed evolving evidence supporting the safety and efficacy of salpingectomy, particularly in high-risk populations. However, the ethical landscape surrounding the procedure necessitates a delicate balance between the potential benefits of cancer prevention and the impact on fertility, emphasizing the importance of informed decision-making and equitable access to preventive care. As salpingectomy emerges as a potential addition to clinical guidelines, clinicians must engage in thorough preoperative counseling, conduct nuanced risk-benefit assessments, and integrate emerging evidence into comprehensive reproductive health discussions. The future of ovarian cancer prevention hinges on continued research, technological innovation, and collaborative efforts, ensuring a holistic and evidence-based approach that holds the promise of a future where the incidence of ovarian cancer is not only treatable but also preventable.

## References

[1] Momenimovahed Z, Tiznobaik A, Taheri S, Salehiniya H (2019). Ovarian cancer in the world: epidemiology and risk factors. Int J Womens Health.

[2] Ferrell B, Cullinane CA, Ervine K, Melancon C, Uman GC, Juarez G (2005). Perspectives on the impact of ovarian cancer: women's views of quality of life. Oncol Nurs Forum.

[3] Kahn RM, Gordhandas S, Godwin K, Stone RL, Worley MJ Jr, Lu KH, Long Roche KC (2023). Salpingectomy for the primary prevention of ovarian cancer: a systematic review. JAMA Surg.

[4] staff CRC (2023). Addressing Ovarian Cancer’s Unique Challenges. American Association for Cancer Research.

[5] (2023). Ovarian Cancer Statistics | How Common is Ovarian Cancer. https://www.cancer.org/cancer/types/ovarian-cancer/about/key-statistics.html.

[6] Forstner R (2020). Early detection of ovarian cancer. Eur Radiol.

[7] Bast RC, Han CY, Lu Z, Lu KH (2021). Next steps in the early detection of ovarian cancer. Commun Med (Lond).

[8] Johnson N, van Voorst S, Sowter MC, Strandell A, Mol BW (2010). Surgical treatment for tubal disease in women due to undergo in vitro fertilisation. Cochrane Database Syst Rev.

[9] Hill CJ, Fakhreldin M, Maclean A (2020). Endometriosis and the fallopian tubes: theories of origin and clinical implications. J Clin Med.

[10] (2023). Opportunistic Salpingectomy as a Strategy for Epithelial Ovarian Cancer Prevention. https://www.acog.org/clinical/clinical-guidance/committee-opinion/articles/2019/04/opportunistic-salpingectomy-as-a-strategy-for-epithelial-ovarian-cancer-prevention.

[11] Sivalingam VN, Duncan WC, Kirk E, Shephard LA, Horne AW (2011). Diagnosis and management of ectopic pregnancy. J Fam Plann Reprod Health Care.

[12] (2023). Salpingectomy. https://www.hopkinsmedicine.org/health/treatment-tests-and-therapies/salpingectomy.

[13] Messinger LB, Alford CE, Csokmay JM, Henne MB, Mumford SL, Segars JH, Armstrong AY (2015). Cost and efficacy comparison of in vitro fertilization and tubal anastomosis for women after tubal ligation. Fertil Steril.

[14] (2023). Salpingectomy: What to Expect from Surgery and Recovery. https://www.medicalnewstoday.com/articles/327146.

[15] Corzo C, Iniesta MD, Patrono MG, Lu KH, Ramirez PT (2017). Role of fallopian tubes in the development of ovarian cancer. J Minim Invasive Gynecol.

[16] Foulkes WD (2013). Preventing ovarian cancer by salpingectomy. Curr Oncol.

[17] Falconer H, Yin L, Grönberg H, Altman D (2015). Ovarian cancer risk after salpingectomy: a nationwide population-based study. J Natl Cancer Inst.

[18] Nakayama K, Nakayama N, Katagiri H, Miyazaki K (2012). Mechanisms of ovarian cancer metastasis: biochemical pathways. Int J Mol Sci.

[19] Wu J, Zhou T, Wang Y, Jiang Y, Wang Y (2021). Mechanisms and advances in anti-ovarian cancer with natural plants component. Molecules.

[20] Yoshihara M, Iyoshi S, Mogi K (2023). Ovarian cancer: Novel mechanisms and therapeutic targets regarding the microenvironment in the abdominal cavity. J Obstet Gynaecol Res.

[21] Hanley GE, Pearce CL, Talhouk A (2022). Outcomes from opportunistic salpingectomy for ovarian cancer prevention. JAMA Netw Open.

[22] Marth C, Abreu MH, Andersen KK (2022). Real-life data on treatment and outcomes in advanced ovarian cancer: an observational, multinational cohort study (RESPONSE trial). Cancer.

[23] (2023). Ovarian Cancer Clinical Trials - Mayo Clinic Research. https://www.mayo.edu/research/clinical-trials/diseases-conditions/ovarian-cancer/.

[24] (2023). Ovarian Cancer Clinical Trials - NCI. https://www.cancer.gov/research/participate/clinical-trials/disease/ovarian-cancer.

[25] Menon U, Gentry-Maharaj A, Burnell M (2021). Ovarian cancer population screening and mortality after long-term follow-up in the UK Collaborative Trial of Ovarian Cancer Screening (UKCTOCS): a randomised controlled trial. Lancet.

[26] (2023). Trial’s Long-Term Follow-Up Data Shows No Difference in Overall Survival Among Ovarian Cancer Patients Who Did and Did Not Receive PARP Inhibitor Maintenance Therapy. https://www.newswise.com/articles/trial-s-long-term-follow-up-data-shows-no-difference-in-overall-survival-among-ovarian-cancer-patients-who-did-and-did-not-receive-parp-inhibitor-maintenance-therapy.

[27] (2023). Outpatient Laparoscopic Salpingectomy. https://outpatienthysterectomy.com/procedure/outpatient-laparoscopic-salpingectomy/.

[28] Lee PS, Bland A, Valea FA, Havrilesky LJ, Berchuck A, Secord AA (2009). Robotic-assisted laparoscopic gynecologic procedures in a fellowship training program. JSLS.

[29] Carugno J, Fatehi M (2024). Abdominal Hysterectomy. https://pubmed.ncbi.nlm.nih.gov/33232036/.

[30] Miranda CS, Carvajal AR (2003). Complications of operative gynecological laparoscopy. JSLS.

[31] Lone PA, Wani NA, Ain QU, Heer A, Devi R, Mahajan S (2021). Common postoperative complications after general anesthesia in oral and maxillofacial surgery. Natl J Maxillofac Surg.

[32] Apfelbaum JL, Chen C, Mehta SS, Gan TJ (2003). Postoperative pain experience: results from a national survey suggest postoperative pain continues to be undermanaged. Anesth Analg.

[33] Venturella R, Lico D, Borelli M (2017). 3 to 5 years later: long-term effects of prophylactic bilateral salpingectomy on ovarian function. J Minim Invasive Gynecol.

[34] Shreffler KM, Johnson DR, Scheuble LK (2010). Ethical problems with infertility treatments: attitudes and explanations. Soc Sci J.

[35] Subramaniam A, Blanchard CT, Erickson BK, Szychowski J, Leath CA, Biggio JR, Huh WK (2018). Feasibility of complete salpingectomy compared with standard postpartum tubal ligation at cesarean delivery: a randomized controlled trial. Obstet Gynecol.

[36] Brock DW, Wikler D (2006). Ethical Issues in Resource Allocation, Research, and New Product Development. https://pubmed.ncbi.nlm.nih.gov/21250319/.

[37] Perets R, Drapkin R (2016). It's totally tubular… riding the new wave of ovarian cancer research. Cancer Res.

[38] (2019). ACOG Committee Opinion No. 774: opportunistic salpingectomy as a strategy for epithelial ovarian cancer prevention. Obstet Gynecol.

[39] Daly MB, Dresher CW, Yates MS, Jeter JM, Karlan BY, Alberts DS, Lu KH (2015). Salpingectomy as a means to reduce ovarian cancer risk. Cancer Prev Res (Phila).

[40] Török P, Naem A, Csehely S, Chiantera V, Sleiman Z, Laganà AS (2023). Reproductive outcomes after expectant and surgical management for tubal pregnancy: a retrospective study. Minim Invasive Ther Allied Technol.

[41] Petrucelli N, Daly MB, Pal T (1993). BRCA1- and BRCA2-Associated Hereditary Breast and Ovarian Cancer. https://pubmed.ncbi.nlm.nih.gov/20301425/.

[42] Venkatesh KK, Clark LH, Stamilio DM (2019). Cost-effectiveness of opportunistic salpingectomy vs tubal ligation at the time of cesarean delivery. Am J Obstet Gynecol.

[43] Cohn F (2010). Oncofertility and informed consent: addressing beliefs, values, and future decision making. Cancer Treat Res.

[44] Tomasch G, Lemmerer M, Oswald S (2020). Prophylactic salpingectomy for prevention of ovarian cancer at the time of elective laparoscopic cholecystectomy. Br J Surg.

[45] Farhud DD, Zokaei S (2021). Ethical issues of artificial intelligence in medicine and healthcare. Iran J Public Health.

[46] Lisio MA, Fu L, Goyeneche A, Gao ZH, Telleria C (2019). High-grade serous ovarian cancer: basic sciences, clinical and therapeutic standpoints. Int J Mol Sci.

[47] Long Roche KC, Abu-Rustum NR, Nourmoussavi M, Zivanovic O (2017). Risk-reducing salpingectomy: let us be opportunistic. Cancer.

[48] Cadish LA, Shepherd JP, Barber EL, Ridgeway B (2017). Risks and benefits of opportunistic salpingectomy during vaginal hysterectomy: a decision analysis. Am J Obstet Gynecol.

[49] Giannakeas V, Murji A, Lipscombe LL, Narod SA, Kotsopoulos J (2023). Salpingectomy and the risk of ovarian cancer in Ontario. JAMA Netw Open.

[50] Sharma R, Biedenharn KR, Fedor JM, Agarwal A (2013). Lifestyle factors and reproductive health: taking control of your fertility. Reprod Biol Endocrinol.

[51] Committee on the State of the Science in Ovarian Cancer Research; Board on Health Care Services; Institute of Medicine; National Academies of Sciences, Engineering Engineering, and Medicine (2016). Ovarian cancers: evolving paradigms in research and care. https://pubmed.ncbi.nlm.nih.gov/27253000/.

[52] Campo-Engelstein L (2010). For the sake of consistency and fairness: why insurance companies should cover fertility preservation treatment for iatrogenic infertility. Cancer Treat Res.

[53] Peters SA, Woodward M, Jha V, Kennedy S, Norton R (2016). Women's health: a new global agenda. BMJ Glob Health.

[54] Alkire BC, Vincent JR, Meara JG (2015). Benefit-cost analysis for selected surgical interventions in low- and middle-income countries. Essential Surgery: Disease Control Priorities, Third Edition.

[55] Kluge EH (2007). Resource allocation in healthcare: implications of models of medicine as a profession. MedGenMed.

[56] Hasan F, Rannaware A, Choudhari SG (2022). Comparison of public health investments of various countries amid a need for greater transparency: a narrative review. Cureus.

[57] Zadabedini Masouleh T, Etchegary H, Hodgkinson K, Wilson BJ, Dawson L (2023). Beyond sterilization: a comprehensive review on the safety and efficacy of opportunistic salpingectomy as a preventative strategy for ovarian cancer. Curr Oncol.

[58] Brown SR, Gregory WM, Twelves CJ (2011). Designing phase II trials in cancer: a systematic review and guidance. Br J Cancer.

[59] Lieu CH, Tan AC, Leong S, Diamond JR, Eckhardt SG (2013). From bench to bedside: lessons learned in translating preclinical studies in cancer drug development. J Natl Cancer Inst.

[60] Kerr RS (2020). Surgery in the 2020s: Implications of advancing technology for patients and the workforce. Future Healthc J.

